# {μ-*trans*-*N*,*N*′-Bis[2-(2-hydroxy­ethyl­amino)eth­yl]oxamidato(2−)}bis­[picrato­nickel(II)]

**DOI:** 10.1107/S1600536809052933

**Published:** 2009-12-16

**Authors:** Chunliang Tian

**Affiliations:** a Department of Chemistry, Jining University, Shandong 273155, People’s Republic of China

## Abstract

The title complex, [Ni_2_(C_6_H_2_N_3_O_7_)_2_(C_10_H_20_N_4_O_4_)], contains a centrosymmetric binuclear unit in which the oxamide ligand (located on a centre of symmetry) acts in a bis-­tetra­dentate fashion and the picrate anion binds to nickel(II) in a bidentate mode. The Ni^II^ atom displays a distorted octa­hedral coordination with axial elongation. The binuclear mol­ecules are linked by inter­molecular N—H⋯O, O—H⋯O and C—H⋯O hydrogen bonds into a two-dimensonal supra­molecular network extending parallel to (100).

## Related literature

For background to oxamido compounds and their complexes, see: Ruiz *et al.* (1999 ▶); Ojima & Nonoyama (1988 ▶).
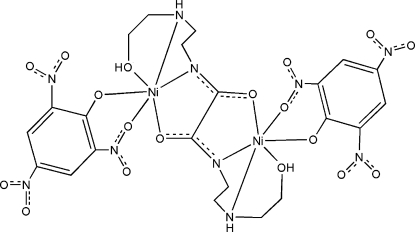


## Experimental

### 

#### Crystal data

[Ni_2_(C_6_H_2_N_3_O_7_)_2_(C_10_H_20_N_4_O_4_)]
*M* *_r_* = 833.93
Triclinic, 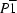

*a* = 7.7893 (16) Å
*b* = 8.1405 (16) Å
*c* = 12.417 (3) Å
α = 98.00 (3)°
β = 99.00 (3)°
γ = 94.36 (3)°
*V* = 766.2 (3) Å^3^
*Z* = 1
Mo *K*α radiation
μ = 1.33 mm^−1^
*T* = 298 K
0.19 × 0.14 × 0.10 mm

#### Data collection

Bruker SMART CCD diffractometer
Absorption correction: multi-scan (*SADABS*; Sheldrick, 1996 ▶) *T*_min_ = 0.786, *T*_max_ = 0.879
4040 measured reflections
2703 independent reflections
2233 reflections with *I* > 2σ(*I*)
*R*_int_ = 0.015

#### Refinement

*R*[*F* ^2^ > 2σ(*F* ^2^)] = 0.035
*wR*(*F* ^2^) = 0.087
*S* = 1.23
2703 reflections
235 parameters
1 restraint
H-atom parameters constrained
Δρ_max_ = 0.39 e Å^−3^
Δρ_min_ = −0.30 e Å^−3^

### 

Data collection: *SMART* (Bruker, 1998 ▶); cell refinement: *SAINT* (Bruker, 1998 ▶); data reduction: *SAINT*; program(s) used to solve structure: *SHELXS97* (Sheldrick, 2008 ▶); program(s) used to refine structure: *SHELXL97* (Sheldrick, 2008 ▶); molecular graphics: *SHELXTL* (Sheldrick, 2008 ▶); software used to prepare material for publication: *SHELXL97* and *PLATON* (Spek, 2009 ▶).

## Supplementary Material

Crystal structure: contains datablocks I, global. DOI: 10.1107/S1600536809052933/si2223sup1.cif

Structure factors: contains datablocks I. DOI: 10.1107/S1600536809052933/si2223Isup2.hkl

Additional supplementary materials:  crystallographic information; 3D view; checkCIF report

## Figures and Tables

**Table 1:** Selected bond lengths (Å)

N1—Ni1	1.903 (3)
N2—Ni1	2.032 (3)
O1—Ni1	1.991 (3)
O2—Ni1	2.537 (3)
O3—Ni1	1.942 (2)
O4—Ni1	2.561 (3)

**Table 2:** Hydrogen-bond geometry (Å, °)

*D*—H⋯*A*	*D*—H	H⋯*A*	*D*⋯*A*	*D*—H⋯*A*
N2—H2*C*⋯O7^i^	0.91	2.15	3.054 (4)	171
O2—H2⋯O1^ii^	0.86	2.34	3.084 (4)	145
C10—H10⋯O5^iii^	0.93	2.49	3.319 (5)	149

Symmetry codes: (i) 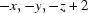; (ii) 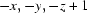; (iii) 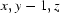.

**Fig. 1.**
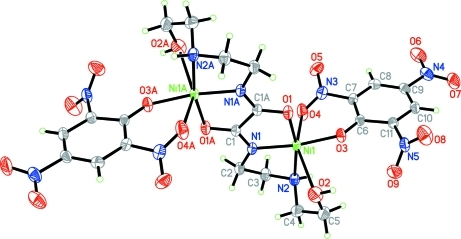
The binuclear complex of (I) with 30% displacement ellipsoids. [Symmetry code: A = -x, -y + 1, -z + 1]

**Fig. 2.**
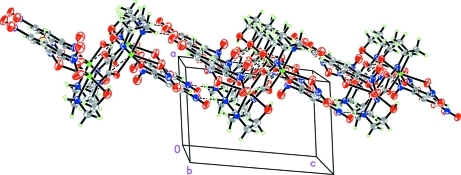
A view of the three-dimensional hydrogen-bonding structure of (I). The H-bonds are shown as dotted lines. [Symmetry codes: i = -x, -y, -z + 2; ii = -x, -y, -z + 1; iii = x, y - 1, z]

## supplementary crystallographic information

### Comment

Oxamido compounds and their complexes have been investigated extensively (Ruiz *et al.*, 1999) by virtue of their bioactivities and the versatile bridging function (Ojima & Nonoyama, 1988). We selected *N,N*'-bis(3-aminopropyl)oxamide as a bridging ligand and picrate as a terminal group to synthesize a new binuclear nickel(II) compound, (I).

The title compound, (I) (Fig. 1), is a binuclear nickel(II) complex containing a total of 52 non-H atoms. Two terminal picrate ligands and a µ_3_-trans-oxamidato bridge with an inversion centre at the mid-point of the C—C bond of the oxamide group. The geometry of each nickel(II) atom is distorted octahedral. For the Ni1, atoms O2 and O4 are in axial positions.  The equatorial plane is composed of three atoms from the oxamide bridge (N1, N2, and O1) and the phenolic oxygen atom (O3) from the picrate ligand. The maximum displacement of the four atoms from the equatorial plane is 0.1117 (15) Å at N1, and the nickel(II) atom lies 0.0524 (8) Å out of the plane. The Ni—N bond lenghts (Table 1) are 1.903 (3) and 2.032 (3)Å, whereas the bond lengths of Ni1—O2 and Ni1—O4 are relatively long and can be considered as a (4+1+1) coordination. As a terminal group, the picrate anion assumes a bidentate mode, forming a six-membered chelate ring on the nickel(II) ion. The dihedral angle between the benzyl ring of the picrate ion and the equatorial plane is 88.28 (3)°.

The crystal structure is stabilized by hydrogen bonding. As shown in Fig. 2, a two-dimensional infinite network is formed via the N—H···O and O—H···O intermolecular hydrogen bonds. The adjacent layers are further connected by non-classical C—H···O hydrogen bonding contacts (Table 2) to form a two-dimensional supramolecular architecture in a zig-zag fashion parallel to the *b,c* plane (Fig. 2).

### Experimental

To a stirred methanol solution (10 ml) containing Ni(pic)_2_^.^6H_2_O (0.1255 g, 0.2 mmol) was added dropwise a ethanol solution (10 ml) of N,N'-bis(N-hydroxyethylaminoethyl)oxamide (0.0262 g, 0.1 mmol) and piperidine (0.0170 g, 0.2 mmol) at room temperature. The mixture was stirred quickly at 323 K for 8 h. The resulting solution was filtered and the filtrate was kept at room temperature. Green crystals suitable for X-ray analysis were obtained from the filtrate by slow evaporation for about two weeks.Yield, 46%, analysis, calculated for C_22_H_24_N_10_O_18_Ni_2_: C 31.69, H, 2.90; N 16.80%; found: C 31.75, H 2.91, N, 16.82%.

### Refinement

H atoms were positioned geometrically [0.93 (CH), 0.97 (CH_2_), 0.85 (OH) and 0.90 (NH)Å] and constrained to ride on their parent atoms with *U*_iso_(H) =1.2*U*_eq_(C/N).

### Crystal data

**Table:**

[Ni_2_(C_6_H_2_N_3_O_7_)_2_(C_10_H_20_N_4_O_4_)]	*Z* = 1
*M**_r_* = 833.93	*F*(000) = 426
Triclinic, *P*1	*D*_x_ = 1.807 Mg m^−^^3^
Hall symbol: -P 1	Mo *K*α radiation, λ = 0.71073 Å
*a* = 7.7893 (16) Å	Cell parameters from 1927 reflections
*b* = 8.1405 (16) Å	θ = 2.5–26.3°
*c* = 12.417 (3) Å	µ = 1.33 mm^−^^1^
α = 98.00 (3)°	*T* = 298 K
β = 99.00 (3)°	Block, green
γ = 94.36 (3)°	0.19 × 0.14 × 0.10 mm
*V* = 766.2 (3) Å^3^	

### Data collection

**Table:**

Bruker SMART CCD diffractometer	2703 independent reflections
Radiation source: fine-focus sealed tube	2233 reflections with *I* > 2σ(*I*)
graphite	*R*_int_ = 0.015
φ and ω scans	θ_max_ = 25.2°, θ_min_ = 1.7°
Absorption correction: multi-scan (*SADABS*; Sheldrick, 1996)	*h* = −9→9
*T*_min_ = 0.786, *T*_max_ = 0.879	*k* = −9→7
4040 measured reflections	*l* = −14→13

### Refinement

**Table:**

Refinement on *F*^2^	Primary atom site location: structure-invariant direct methods
Least-squares matrix: full	Secondary atom site location: difference Fourier map
*R*[*F*^2^ > 2σ(*F*^2^)] = 0.035	Hydrogen site location: inferred from neighbouring sites
*wR*(*F*^2^) = 0.087	H-atom parameters constrained
*S* = 1.23	*w* = 1/[σ^2^(*F*_o_^2^) + (0.0205*P*)^2^ + 0.7682*P*] where *P* = (*F*_o_^2^ + 2*F*_c_^2^)/3
2703 reflections	(Δ/σ)_max_ = 0.001
235 parameters	Δρ_max_ = 0.39 e Å^−^^3^
1 restraint	Δρ_min_ = −0.30 e Å^−^^3^

### Special details

**Table:**

Experimental. a DELU restraint was applied for Ni1 O2 with s.u. 0.002.
Geometry. All esds (except the esd in the dihedral angle between two l.s. planes) are estimated using the full covariance matrix. The cell esds are taken into account individually in the estimation of esds in distances, angles and torsion angles; correlations between esds in cell parameters are only used when they are defined by crystal symmetry. An approximate (isotropic) treatment of cell esds is used for estimating esds involving l.s. planes.
Refinement. Refinement of F^2^ against ALL reflections. The weighted R-factor wR and goodness of fit S are based on F^2^, conventional R-factors R are based on F, with F set to zero for negative F^2^. The threshold expression of F^2^ > 2sigma(F^2^) is used only for calculating R-factors(gt) etc. and is not relevant to the choice of reflections for refinement. R-factors based on F^2^ are statistically about twice as large as those based on F, and R- factors based on ALL data will be even larger.

### Fractional atomic coordinates and isotropic or equivalent isotropic displacement parameters (Å^2^)

**Table:**

	*x*	*y*	*z*	*U*_iso_*/*U*_eq_	
C1	0.0883 (4)	0.5501 (4)	0.5131 (3)	0.0339 (8)	
C2	0.3717 (5)	0.5619 (5)	0.6319 (3)	0.0446 (9)	
H2A	0.3764	0.6824	0.6485	0.054*	
H2B	0.4516	0.5339	0.5811	0.054*	
C3	0.4200 (5)	0.4875 (5)	0.7369 (3)	0.0471 (10)	
H3A	0.5460	0.4988	0.7586	0.057*	
H3B	0.3704	0.5470	0.7960	0.057*	
C4	0.4620 (5)	0.2008 (5)	0.6579 (4)	0.0553 (11)	
H4A	0.5691	0.1901	0.7069	0.066*	
H4B	0.4930	0.2524	0.5968	0.066*	
C5	0.3688 (6)	0.0319 (5)	0.6149 (4)	0.0609 (12)	
H5A	0.4466	−0.0410	0.5831	0.073*	
H5B	0.3266	−0.0170	0.6740	0.073*	
C6	−0.0763 (4)	0.1046 (4)	0.7864 (3)	0.0334 (8)	
C7	−0.1144 (5)	0.2293 (4)	0.8705 (3)	0.0375 (8)	
C8	−0.1927 (5)	0.1921 (5)	0.9576 (3)	0.0418 (9)	
H8	−0.2158	0.2773	1.0098	0.050*	
C9	−0.2359 (5)	0.0297 (5)	0.9669 (3)	0.0412 (9)	
C10	−0.2086 (5)	−0.0995 (5)	0.8879 (3)	0.0415 (9)	
H10	−0.2417	−0.2098	0.8935	0.050*	
C11	−0.1322 (5)	−0.0607 (4)	0.8020 (3)	0.0361 (8)	
N1	0.1953 (4)	0.4904 (4)	0.5843 (2)	0.0369 (7)	
N2	0.3526 (4)	0.3088 (4)	0.7183 (2)	0.0415 (7)	
H2C	0.3490	0.2754	0.7849	0.050*	
N3	−0.0728 (5)	0.4047 (4)	0.8665 (3)	0.0477 (8)	
N4	−0.3140 (4)	−0.0112 (6)	1.0595 (3)	0.0551 (9)	
N5	−0.1030 (5)	−0.1992 (4)	0.7211 (3)	0.0502 (8)	
O1	−0.1140 (3)	0.3225 (3)	0.5345 (2)	0.0428 (6)	
O2	0.2266 (4)	0.0540 (4)	0.5331 (2)	0.0666 (8)	
H2	0.1526	−0.0338	0.5205	0.080*	
O3	−0.0095 (3)	0.1265 (3)	0.70232 (19)	0.0436 (6)	
O4	0.0475 (5)	0.4475 (4)	0.8204 (3)	0.0690 (9)	
O5	−0.1590 (4)	0.5052 (4)	0.9118 (3)	0.0659 (9)	
O6	−0.3605 (5)	0.1016 (5)	1.1207 (3)	0.0776 (10)	
O7	−0.3329 (4)	−0.1582 (5)	1.0716 (3)	0.0751 (10)	
O8	−0.2239 (5)	−0.3026 (4)	0.6812 (3)	0.0860 (11)	
O9	0.0423 (5)	−0.2056 (4)	0.6993 (3)	0.0839 (11)	
Ni1	0.10416 (6)	0.29778 (6)	0.63579 (4)	0.03242 (15)	

### Atomic displacement parameters (Å^2^)

**Table:**

	*U*^11^	*U*^22^	*U*^33^	*U*^12^	*U*^13^	*U*^23^
C1	0.042 (2)	0.0292 (18)	0.0307 (18)	0.0014 (15)	0.0064 (15)	0.0059 (14)
C2	0.044 (2)	0.040 (2)	0.049 (2)	−0.0028 (18)	0.0027 (18)	0.0139 (18)
C3	0.050 (2)	0.042 (2)	0.044 (2)	−0.0062 (18)	−0.0010 (18)	0.0075 (18)
C4	0.047 (2)	0.053 (3)	0.066 (3)	0.008 (2)	0.007 (2)	0.013 (2)
C5	0.075 (3)	0.045 (3)	0.069 (3)	0.011 (2)	0.025 (3)	0.014 (2)
C6	0.0320 (18)	0.0347 (19)	0.0329 (19)	0.0005 (15)	0.0000 (15)	0.0111 (15)
C7	0.042 (2)	0.033 (2)	0.0366 (19)	0.0030 (16)	0.0029 (16)	0.0090 (16)
C8	0.039 (2)	0.051 (2)	0.034 (2)	0.0085 (18)	0.0046 (16)	0.0037 (17)
C9	0.037 (2)	0.057 (3)	0.0332 (19)	0.0033 (18)	0.0075 (16)	0.0150 (18)
C10	0.039 (2)	0.043 (2)	0.043 (2)	−0.0024 (17)	0.0038 (17)	0.0175 (18)
C11	0.038 (2)	0.037 (2)	0.0332 (19)	0.0017 (16)	0.0066 (15)	0.0068 (15)
N1	0.0415 (17)	0.0315 (16)	0.0375 (16)	−0.0006 (13)	0.0038 (14)	0.0102 (13)
N2	0.0489 (19)	0.0416 (18)	0.0341 (16)	0.0012 (15)	0.0034 (14)	0.0122 (14)
N3	0.063 (2)	0.0400 (19)	0.0382 (18)	0.0034 (17)	0.0061 (17)	0.0048 (15)
N4	0.047 (2)	0.083 (3)	0.0378 (19)	−0.001 (2)	0.0070 (16)	0.020 (2)
N5	0.065 (2)	0.0384 (19)	0.050 (2)	−0.0060 (18)	0.0203 (18)	0.0107 (16)
O1	0.0475 (15)	0.0405 (15)	0.0396 (14)	−0.0067 (12)	0.0017 (12)	0.0162 (11)
O2	0.075 (2)	0.0558 (18)	0.064 (2)	−0.0065 (15)	0.0163 (17)	−0.0078 (15)
O3	0.0592 (17)	0.0378 (15)	0.0348 (14)	−0.0045 (12)	0.0134 (12)	0.0086 (11)
O4	0.101 (3)	0.0412 (17)	0.067 (2)	−0.0132 (17)	0.0391 (19)	0.0013 (15)
O5	0.081 (2)	0.0470 (18)	0.071 (2)	0.0230 (17)	0.0149 (18)	0.0048 (15)
O6	0.080 (2)	0.108 (3)	0.0499 (19)	0.004 (2)	0.0307 (18)	0.0088 (19)
O7	0.083 (2)	0.091 (3)	0.063 (2)	0.001 (2)	0.0255 (18)	0.0423 (19)
O8	0.097 (3)	0.068 (2)	0.079 (2)	−0.030 (2)	0.018 (2)	−0.0194 (19)
O9	0.085 (3)	0.054 (2)	0.122 (3)	0.0086 (18)	0.060 (2)	−0.0012 (19)
Ni1	0.0384 (3)	0.0306 (2)	0.0281 (2)	−0.00288 (18)	0.00314 (18)	0.01040 (17)

### Geometric parameters (Å, °)

**Table:**

C1—O1^i^	1.282 (4)	C8—C9	1.364 (5)
C1—N1	1.289 (4)	C8—H8	0.9300
C1—C1^i^	1.510 (7)	C9—C10	1.387 (5)
C2—N1	1.451 (5)	C9—N4	1.449 (5)
C2—C3	1.520 (5)	C10—C11	1.363 (5)
C2—H2A	0.9700	C10—H10	0.9300
C2—H2B	0.9700	C11—N5	1.458 (5)
C3—N2	1.482 (5)	N1—Ni1	1.903 (3)
C3—H3A	0.9700	N2—Ni1	2.032 (3)
C3—H3B	0.9700	N2—H2C	0.9100
C4—N2	1.479 (5)	N3—O4	1.225 (4)
C4—C5	1.492 (6)	N3—O5	1.229 (4)
C4—H4A	0.9700	N4—O6	1.223 (5)
C4—H4B	0.9700	N4—O7	1.227 (5)
C5—O2	1.421 (5)	N5—O9	1.208 (4)
C5—H5A	0.9700	N5—O8	1.208 (4)
C5—H5B	0.9700	O1—C1^i^	1.282 (4)
C6—O3	1.266 (4)	O1—Ni1	1.991 (3)
C6—C11	1.431 (5)	O2—Ni1	2.537 (3)
C6—C7	1.433 (5)	O2—H2	0.8634
C7—C8	1.380 (5)	O3—Ni1	1.942 (2)
C7—N3	1.450 (5)	O4—Ni1	2.561 (3)
			
O1^i^—C1—N1	128.9 (3)	C10—C11—C6	125.0 (3)
O1^i^—C1—C1^i^	119.2 (4)	C10—C11—N5	117.1 (3)
N1—C1—C1^i^	111.9 (4)	C6—C11—N5	117.9 (3)
N1—C2—C3	106.0 (3)	C1—N1—C2	126.0 (3)
N1—C2—H2A	110.5	C1—N1—Ni1	115.6 (2)
C3—C2—H2A	110.5	C2—N1—Ni1	118.3 (2)
N1—C2—H2B	110.5	C4—N2—C3	112.9 (3)
C3—C2—H2B	110.5	C4—N2—Ni1	112.5 (2)
H2A—C2—H2B	108.7	C3—N2—Ni1	105.4 (2)
N2—C3—C2	109.9 (3)	C4—N2—H2C	108.6
N2—C3—H3A	109.7	C3—N2—H2C	108.6
C2—C3—H3A	109.7	Ni1—N2—H2C	108.6
N2—C3—H3B	109.7	O4—N3—O5	122.6 (4)
C2—C3—H3B	109.7	O4—N3—C7	119.3 (3)
H3A—C3—H3B	108.2	O5—N3—C7	118.0 (3)
N2—C4—C5	111.5 (3)	O6—N4—O7	123.2 (4)
N2—C4—H4A	109.3	O6—N4—C9	118.6 (4)
C5—C4—H4A	109.3	O7—N4—C9	118.2 (4)
N2—C4—H4B	109.3	O9—N5—O8	123.6 (4)
C5—C4—H4B	109.3	O9—N5—C11	117.9 (3)
H4A—C4—H4B	108.0	O8—N5—C11	118.5 (4)
O2—C5—C4	106.6 (4)	C1^i^—O1—Ni1	108.8 (2)
O2—C5—H5A	110.4	C5—O2—Ni1	99.8 (2)
C4—C5—H5A	110.4	C5—O2—H2	109.0
O2—C5—H5B	110.4	Ni1—O2—H2	110.6
C4—C5—H5B	110.4	C6—O3—Ni1	142.1 (2)
H5A—C5—H5B	108.6	N3—O4—Ni1	124.3 (2)
O3—C6—C11	119.7 (3)	N1—Ni1—O3	170.56 (12)
O3—C6—C7	127.8 (3)	N1—Ni1—O1	84.45 (11)
C11—C6—C7	112.5 (3)	O3—Ni1—O1	93.01 (11)
C8—C7—C6	123.2 (3)	N1—Ni1—N2	82.75 (12)
C8—C7—N3	116.3 (3)	O3—Ni1—N2	100.22 (12)
C6—C7—N3	120.5 (3)	O1—Ni1—N2	166.66 (11)
C9—C8—C7	119.7 (4)	N1—Ni1—O2	105.46 (11)
C9—C8—H8	120.1	O3—Ni1—O2	83.96 (11)
C7—C8—H8	120.1	O1—Ni1—O2	103.16 (11)
C8—C9—C10	121.2 (3)	N2—Ni1—O2	76.77 (12)
C8—C9—N4	120.3 (4)	N1—Ni1—O4	96.75 (11)
C10—C9—N4	118.5 (4)	O3—Ni1—O4	74.84 (10)
C11—C10—C9	118.3 (3)	O1—Ni1—O4	101.91 (12)
C11—C10—H10	120.8	N2—Ni1—O4	83.37 (12)
C9—C10—H10	120.8	O2—Ni1—O4	147.80 (11)
			
N1—C2—C3—N2	39.7 (4)	O5—N3—O4—Ni1	−138.5 (3)
N2—C4—C5—O2	66.6 (4)	C7—N3—O4—Ni1	42.8 (5)
O3—C6—C7—C8	178.0 (3)	C1—N1—Ni1—O3	−74.6 (8)
C11—C6—C7—C8	1.4 (5)	C2—N1—Ni1—O3	102.0 (7)
O3—C6—C7—N3	−1.3 (6)	C1—N1—Ni1—O1	0.2 (3)
C11—C6—C7—N3	−177.9 (3)	C2—N1—Ni1—O1	176.8 (3)
C6—C7—C8—C9	0.6 (5)	C1—N1—Ni1—N2	176.4 (3)
N3—C7—C8—C9	179.9 (3)	C2—N1—Ni1—N2	−7.0 (3)
C7—C8—C9—C10	−2.4 (5)	C1—N1—Ni1—O2	102.3 (3)
C7—C8—C9—N4	178.4 (3)	C2—N1—Ni1—O2	−81.1 (3)
C8—C9—C10—C11	2.0 (5)	C1—N1—Ni1—O4	−101.2 (3)
N4—C9—C10—C11	−178.8 (3)	C2—N1—Ni1—O4	75.4 (3)
C9—C10—C11—C6	0.2 (6)	C6—O3—Ni1—N1	−25.6 (10)
C9—C10—C11—N5	179.2 (3)	C6—O3—Ni1—O1	−99.7 (4)
O3—C6—C11—C10	−178.8 (3)	C6—O3—Ni1—N2	82.1 (4)
C7—C6—C11—C10	−1.8 (5)	C6—O3—Ni1—O2	157.4 (4)
O3—C6—C11—N5	2.3 (5)	C6—O3—Ni1—O4	1.9 (4)
C7—C6—C11—N5	179.2 (3)	C1^i^—O1—Ni1—N1	−0.3 (2)
O1^i^—C1—N1—C2	3.4 (6)	C1^i^—O1—Ni1—O3	170.6 (2)
C1^i^—C1—N1—C2	−176.4 (3)	C1^i^—O1—Ni1—N2	−16.7 (6)
O1^i^—C1—N1—Ni1	179.7 (3)	C1^i^—O1—Ni1—O2	−104.9 (2)
C1^i^—C1—N1—Ni1	−0.1 (5)	C1^i^—O1—Ni1—O4	95.5 (2)
C3—C2—N1—C1	160.4 (3)	C4—N2—Ni1—N1	−95.1 (3)
C3—C2—N1—Ni1	−15.9 (4)	C3—N2—Ni1—N1	28.3 (2)
C5—C4—N2—C3	−165.1 (3)	C4—N2—Ni1—O3	93.9 (3)
C5—C4—N2—Ni1	−46.0 (4)	C3—N2—Ni1—O3	−142.6 (2)
C2—C3—N2—C4	78.4 (4)	C4—N2—Ni1—O1	−78.6 (6)
C2—C3—N2—Ni1	−44.8 (4)	C3—N2—Ni1—O1	44.8 (6)
C8—C7—N3—O4	153.4 (4)	C4—N2—Ni1—O2	12.7 (2)
C6—C7—N3—O4	−27.3 (5)	C3—N2—Ni1—O2	136.1 (2)
C8—C7—N3—O5	−25.4 (5)	C4—N2—Ni1—O4	167.2 (3)
C6—C7—N3—O5	154.0 (3)	C3—N2—Ni1—O4	−69.4 (2)
C8—C9—N4—O6	9.2 (5)	C5—O2—Ni1—N1	98.6 (2)
C10—C9—N4—O6	−170.0 (4)	C5—O2—Ni1—O3	−81.9 (2)
C8—C9—N4—O7	−172.0 (4)	C5—O2—Ni1—O1	−173.6 (2)
C10—C9—N4—O7	8.8 (5)	C5—O2—Ni1—N2	20.1 (2)
C10—C11—N5—O9	−127.5 (4)	C5—O2—Ni1—O4	−33.3 (3)
C6—C11—N5—O9	51.5 (5)	N3—O4—Ni1—N1	147.0 (3)
C10—C11—N5—O8	51.1 (5)	N3—O4—Ni1—O3	−28.6 (3)
C6—C11—N5—O8	−129.9 (4)	N3—O4—Ni1—O1	61.3 (3)
C4—C5—O2—Ni1	−47.1 (3)	N3—O4—Ni1—N2	−131.1 (3)
C11—C6—O3—Ni1	−173.9 (3)	N3—O4—Ni1—O2	−79.2 (4)
C7—C6—O3—Ni1	9.7 (6)		

Symmetry codes: (i) −*x*, −*y*+1, −*z*+1.

### Hydrogen-bond geometry (Å, °)

**Table:**

*D*—H···*A*	*D*—H	H···*A*	*D*···*A*	*D*—H···*A*
N2—H2C···O7^ii^	0.91	2.15	3.054 (4)	171
O2—H2···O1^iii^	0.86	2.34	3.084 (4)	145
C10—H10···O5^iv^	0.93	2.49	3.319 (5)	149

Symmetry codes: (ii) −*x*, −*y*, −*z*+2; (iii) −*x*, −*y*, −*z*+1; (iv) *x*, *y*−1, *z*.
